# Thoracic disk herniation diagnosed with an upper body traction procedure in the sitting position

**DOI:** 10.1002/jgf2.405

**Published:** 2020-12-14

**Authors:** Kosuke Ishizuka, Daiki Yokokawa, Masatomi Ikusaka

**Affiliations:** ^1^ Department of General Medicine Chiba University Hospital Chiba Japan

**Keywords:** thoracic disk herniation, upper body traction procedure

## Abstract

A 51‐year‐old man experienced sudden abdominal pain from the umbilicus to the right flank 5 days before his hospital visit. His abdominal pain disappeared when the examiner lifted his upper body in the sitting position. MRI revealed posterior intervertebral disc protrusion in the right paramedian region at the 9th/10th thoracic vertebrae. With the treatment, it is reported that traction is the appropriate initial approach for spine radiculopathy. Improvement with upper body traction performed in this case, which is a previously unreported maneuver, appears to be useful for diagnosis because it eliminates the influence of gravity and reduces intradiscal pressure.

## Graphical abstract

Abdominal pain originating from the spine is often overlooked because most intervertebral disk herniation occurs at the lumber vertebra that produce buttock and/or leg pain. In thoracic disk herniation, the radicular pain is aggravated by posture, especially forward‐bending and sitting position, both of which increases the intradiscal pressure. Also, improvement with upper body traction, unreported maneuver, appears to be useful for diagnosis because it eliminates the influence of gravity and reduces intradiscal pressure.

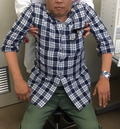

A 51‐year‐old man experienced sudden abdominal pain from the umbilicus to the right flank, when he did desk work 5 days before his hospital visit. The pain worsened in the sitting and forward‐bending positions, but subsided in the standing position. His past medical history was only remarkable of polycythemia vera for which he was on 100mg/day of oral aspirin. He has smoked 1 pack per day of cigarettes for over 25 years. The pain distribution corresponded to the Th10 dermatome, but physical examination showed no rash or tenderness on the trunk including the abdomen. Neurological examination was unremarkable. The Kemp's test was negative. His abdominal pain disappeared completely when the examiner lifted his upper body in the sitting position (Figure 1). Herpes zoster should be differentiated from acute onset lateral abdominal pain. However, the exacerbations and remissions by posture cannot be explained. We considered radicular pain from the thoracic spine, because both of forward‐bending and sitting position are the postures that increase the intradiscal pressure, and because the pain distribution corresponded to the Th10 dermatome. Thoracic spine MRI revealed posterior intervertebral disc protrusion in the right paramedian region at the 9th/10th thoracic vertebrae (Figure 2). His abdominal pain was alleviated with oral administration of pregabalin 150 mg/day.

**Figure 1:**
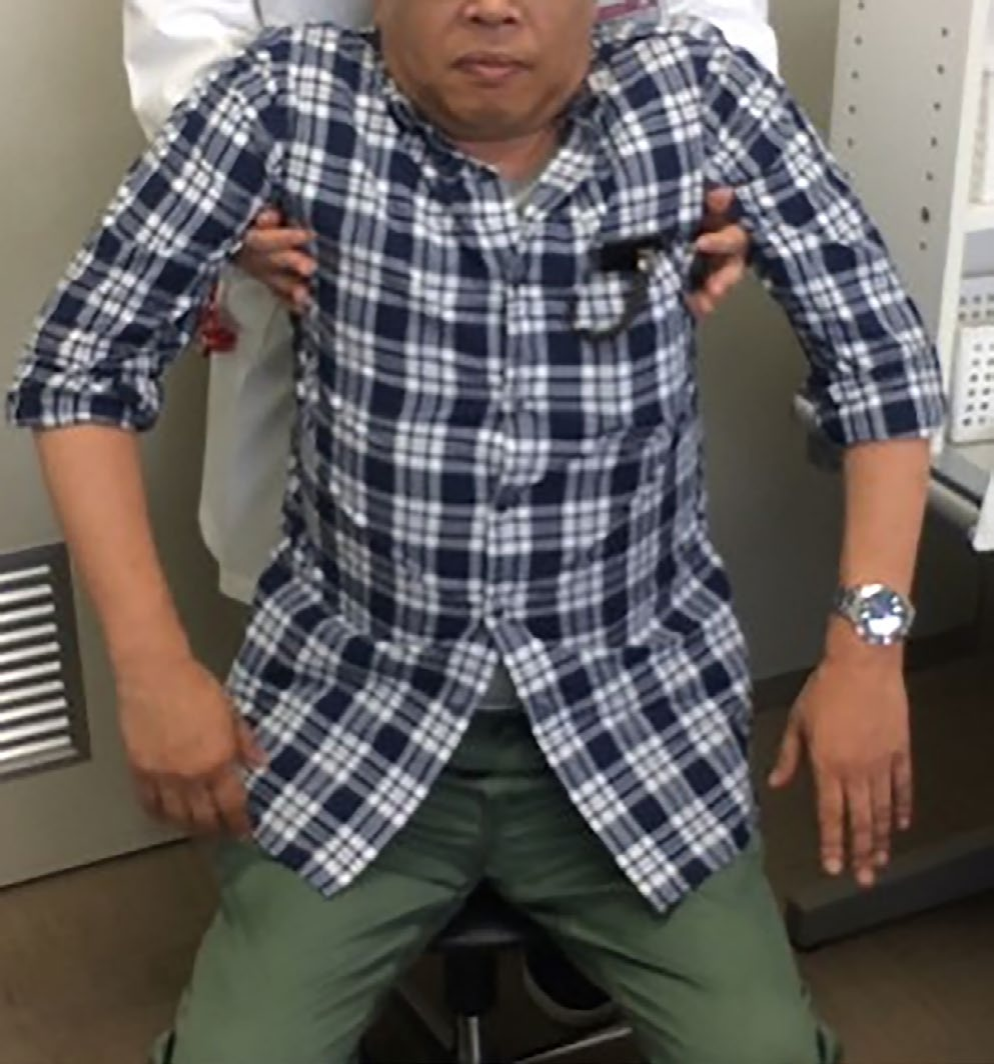
Upper body traction maneuver: his abdominal pain disappeared completely when the examiner lifted his upper body in the sitting position

**Figure 2:**
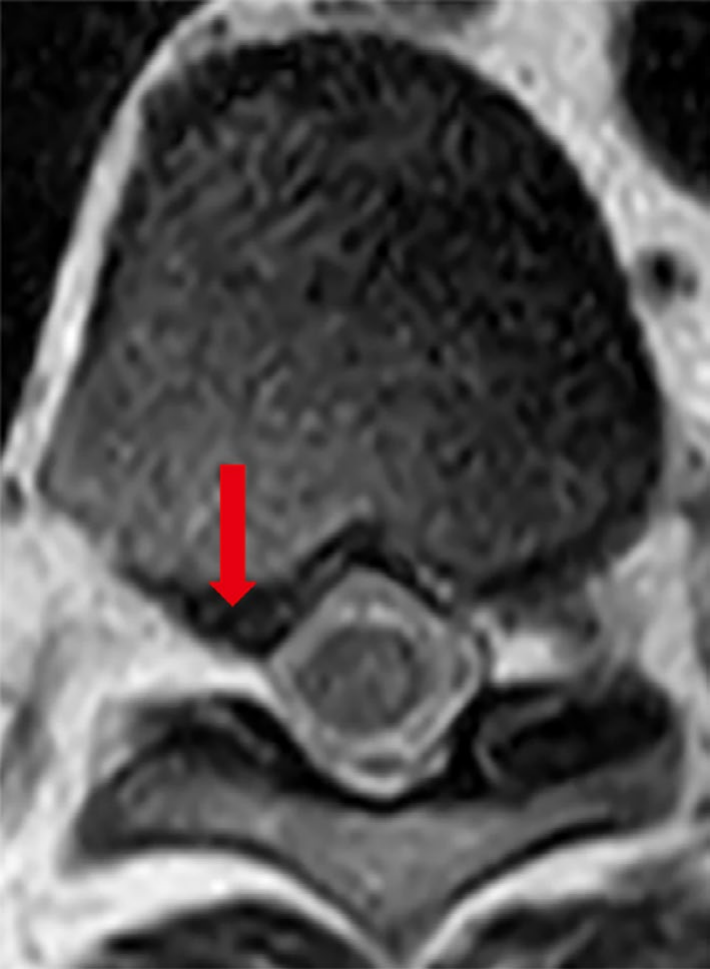
Thoracic spine MRI revealed posterior intervertebral disk protrusion in the right paramedian region at the 9th/10th thoracic vertebrae

An abdominal wall source can be found in 30% of cases of chronic abdominal pain.^1^ Chronic abdominal wall pain is often misinterpreted as arising from a visceral source, frequently leading to inappropriate diagnostic tests, unsatisfactory treatments, and considerable cost.^2^ The most common cause is entrapment of an anterior branch of one or more thoracic intercostal nerves; myofascial pain and radiculopathy are less frequent.^2^ Abdominal pain originating from the spine is often overlooked because most intervertebral disk herniation occurs at the lumber vertebra that produce buttock and/or leg pain.^3^ Thoracic disk herniation accounts for less than 1% of all disk protrusions.^4^ Arce, *et al.* (1985) reported that 75% of cases occurred below Th8, which is a large range of motion, whereas 3% of cases occurred between Th1 and Th2, and less than 1% occurred between Th2 and Th3 Trauma is also considered to be a major factor.^5^ Up to 25% of patients have been found to report a history of trauma.^2^ Stillerman, et al^2^ reported that symptoms of the thoracic disc herniation included radicular pain (76%), myelopathy (61%), and bowel/bladder dysfunction (24%).^3^ Lara, et al. (2012) reported that the radicular pain was aggravated by posture in 72% of the subjects, especially forward‐bending and sitting position, both of which increases the intradiscal pressure.^2^, ^6^ He also reported that physical examination showed abnormalities in the pain and temperature sensation in 61% of the patients.^2^ The literature supporting the use of Kemp's test is limited and indicates that it has poor diagnostic accuracy.^7^ The test cannot be ruled out even if the result is negative. With the treatment, it is reported that traction is the appropriate initial approach and has been proposed for cervical and lumbar radiculopathy.^8^ In addition, thoracic traction has been reported as one of the spine tractions.^9^ Improvement with upper body traction performed in this case, which is a previously unreported maneuver, appears to be also useful for diagnosis because it eliminates the influence of gravity and reduces intradiscal pressure.

## CONFLICT OF INTEREST

None.

## AUTHOR CONTRIBUTIONS

All authors had access to the data and a role in writing the manuscript.

## INFORMED CONSENT

The patient's written consent was obtained for publication.
